# Identification of an Archaeal Presenilin-Like Intramembrane Protease

**DOI:** 10.1371/journal.pone.0013072

**Published:** 2010-09-29

**Authors:** Celia Torres-Arancivia, Carolyn M. Ross, Jose Chavez, Zahra Assur, Georgia Dolios, Filippo Mancia, Iban Ubarretxena-Belandia

**Affiliations:** 1 Department of Structural and Chemical Biology, Mount Sinai School of Medicine, New York, New York, United States of America; 2 The Graduate Center, City University of New York, New York, New York, United States of America; 3 Department of Biochemistry and Molecular Biophysics, Columbia University, New York, New York, United States of America; 4 Department of Human Genetics, Mount Sinai School of Medicine, New York, New York, United States of America; 5 Department of Physiology and Cellular Biophysics, Columbia University, New York, New York, United States of America; Massachusetts Institute of Technology, United States of America

## Abstract

**Background:**

The GXGD-type diaspartyl intramembrane protease, presenilin, constitutes the catalytic core of the γ-secretase multi-protein complex responsible for activating critical signaling cascades during development and for the production of β-amyloid peptides (Aβ) implicated in Alzheimer's disease. The only other known GXGD-type diaspartyl intramembrane proteases are the eukaryotic signal peptide peptidases (SPPs). The presence of presenilin-like enzymes outside eukaryots has not been demonstrated. Here we report the existence of presenilin-like GXGD-type diaspartyl intramembrane proteases in archaea.

**Methodology and Principal Findings:**

We have employed *in vitro* activity assays to show that MCMJR1, a polytopic membrane protein from the archaeon *Methanoculleus marisnigri* JR1, is an intramembrane protease bearing the signature YD and GXGD catalytic motifs of presenilin-like enzymes. Mass spectrometry analysis showed MCMJR1 could cleave model intramembrane protease substrates at several sites within their transmembrane region. Remarkably, MCMJR1 could also cleave substrates derived from the β-amyloid precursor protein (APP) without the need of protein co-factors, as required by presenilin. Two distinct cleavage sites within the transmembrane domain of APP could be identified, one of which coincided with Aβ40, the predominant site processed by γ-secretase. Finally, an established presenilin and SPP transition-state analog inhibitor could inhibit MCMJR1.

**Conclusions and Significance:**

Our findings suggest that a primitive GXGD-type diaspartyl intramembrane protease from archaea can recapitulate key biochemical properties of eukaryotic presenilins and SPPs. MCMJR1 promises to be a more tractable, simpler system for in depth structural and mechanistic studies of GXGD-type diaspartyl intramembrane proteases.

## Introduction

Regulated intramembrane proteolysis is an ancient mechanism to control cell metabolism, differentiation and development in organisms ranging from bacteria to humans [1]. In intramembrane proteolysis, single-pass membrane proteins are cleaved within their transmembrane domain (TMD) to liberate soluble fragments that can then act as molecular effectors. Examples include the release of transcriptional activators in the Notch [2] and ErbB-4 [3] signaling cascades; the production of the neuropathogenic β-amyloid peptides (Aβ) [4]; the liberation of cellular growth factors [5]; and the regulation of cholesterol biosynthesis [6]. The intramembrane-cleaving proteases (known as i-CLiPs) constitute a novel class of integral membrane proteins. In analogy to their water-soluble counterparts, i-CLiPs can be divided into aspartic proteases, metalloproteases and serine proteases [7]. GXGD-type diaspartyl intramembrane proteases are arguably the most relevant i-CLiPs from the perspective of human biology and health [8], [9].

**Figure 1:**
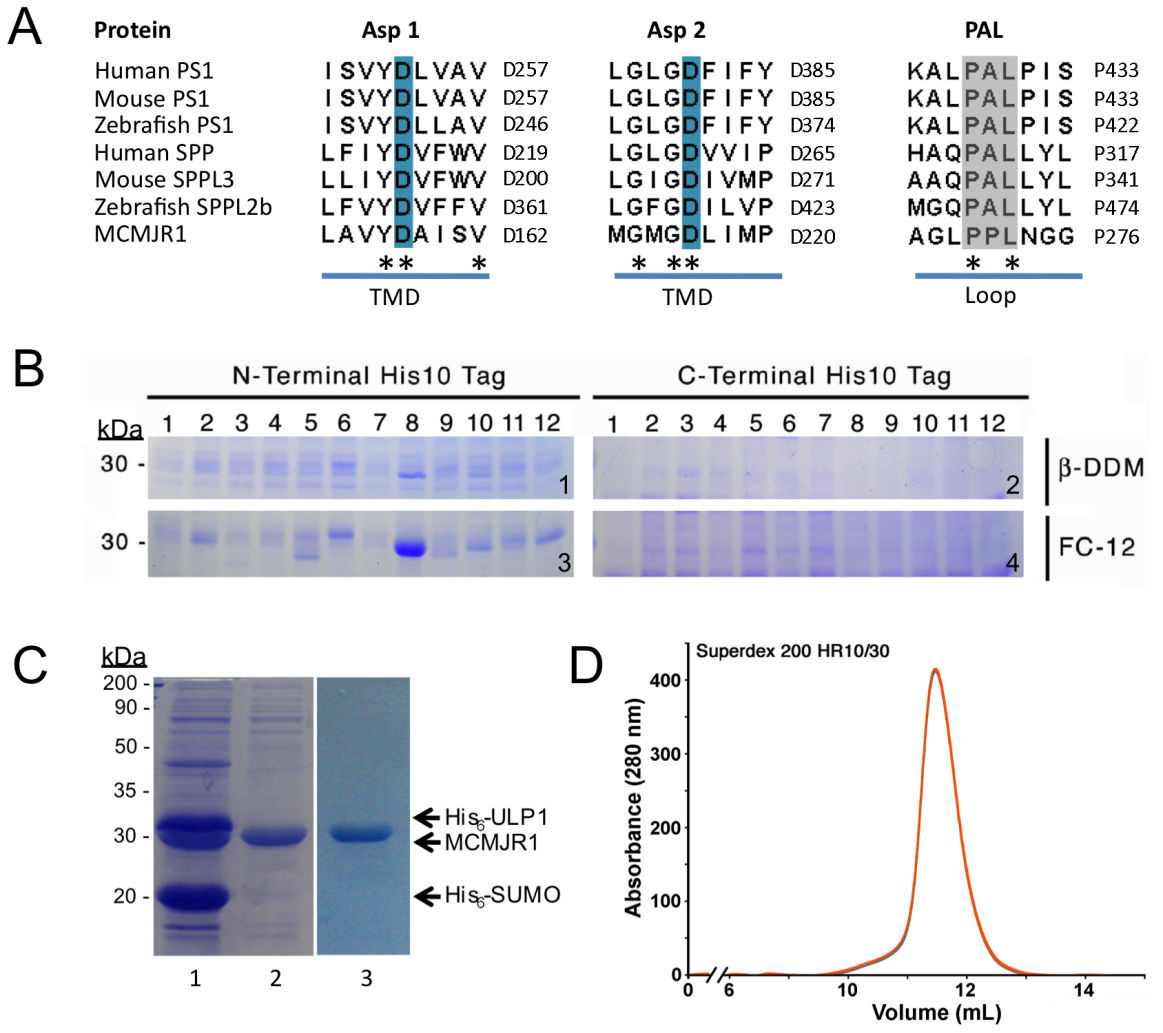
Identification of GXGD-type diaspartyl intramembrane proteases in archaea. **A.** Multiple sequence alignment (CLUSTAL) of presenilin 1 (PS1) homologs, signal peptide peptidase (SPP) homologs and MCMJR1 around the TMD regions encompassing the two catalytic aspartates (Asp 1 and Asp 2) and the loop region containing the C-terminal PAL motif. A star marks absolutely conserved amino acids. The catalytic aspartates (blue) and the proline in the PAL motif (gray) are numbered. **B.** Genomic expansion of archaeal GXGD-type diaspartyl intramembrane protease targets. The target proteins purified from a 100 mL bacterial culture, bearing either N or C terminal His_10_-tags (Panels **1**, **3** and **2**, **4** respectively) were purified by metal affinity chromatography. Purification was performed in DDM (Panel **1** and **2**) and FC-12 (Panel **3** and **4**). Homologues from 12 different genomes were screened: 1, *Haloarcula marismortui*; 2, *Methanosarcina mazei*; 3, *Archaeoglobus fulgidus*; 4, *Ferroplasma acidarmanus*; 5, *Picrophilus torridus*; 6, *Methanospirillum hungatei*; 7, *Thermoplasma volcanium*; 8, *Methanoculleus marisnigri*; 9, *Thermoplasma acidophilum*; 10, *Methanosarcina barkeri*; 11, *Methanococcoides burtonii*; 12, *Methanosarcina acetivorans*. MCMJR1 corresponds to lane 8. **C.** A coomassie stained 10% SDS-PAGE analysis of MCMJR1 purification. Samples corresponding to purified MCMJR1 incubated with ULP1 protease (lane 1), after re-passing the preparation through Ni-NTA resin (lane 2) and after SEC over a HR200 superdex column (lane 3). The molecular weight marker positions are shown on the left. **D.** Elution profile from MCMJR1 run on a SEC column.

Presenilins are the founding members of GXGD-type diaspartyl intramembrane proteases. These enzymes are human polytopic integral membrane proteins with nine predicted TMDs [10], and with conserved YD and GXGD signature motifs in adjacent TMDs (Fig. 1A) providing the two catalytic aspartate amino acid residues [11]. A third conserved short stretch (PAL motif) is typically present in the C-terminal region of presenilin genes and is also considered to have a functional role [12]. Presenilins received early attention due to genetic studies showing that rare, early-onset autosomal dominant forms of familial Alzheimer's disease (AD) are caused by the inheritance of gene variants of this enzyme [13]. Subsequent studies demonstrated that presenilins constituted the catalytic core of γ-secretase [14], [15], a multi-protein complex [16] composed of presenilin, nicastrin, anterior pharynx defective (APH-1) and presenilin enhancer 2 (PEN-2). To date, γ-secretase has been shown to be responsible for the processing of a growing number of type I integral membrane proteins including APP, APP-like proteins, E-Cadherin, CD44, lipoprotein receptor related protein, Notch, interferon response element and activated transcription factor 6 [17]. As γ-secretase consists of four hydrophobic proteins totaling at least 19 TMDs its structural and functional characterization is particularly challenging [18].

Presenilins were initially considered to be an isolated protein family present only in vertebrates. However in a more recent study, Ponting *et al.* suggested the presence of presenilin-like proteins throughout eukaryotes, in fungi as well as in plants [19]. Shortly after, one of the human candidates was identified as signal peptide peptidase (SPP) and shown to exert intramembrane protease activity on the signal sequence of polymorphic major histocompatibility complex class I molecules [20]. Human SPP is a polytopic integral membrane protein with seven predicted TMDs. Like presenilin, the two catalytic aspartates in SPP are found within the conserved YD and GXGD motifs located in adjacent TMDs [20]. In addition, SPP can be photolabeled by a γ-secretase transition state analog inhibitor, suggesting a possible conservation of active-site structure within the two enzymes [21], and even Aβ modulators affect SPP activity [22]. These findings have provided strong evidence that SPP and presenilin share significant biochemical properties and have promoted the use of SPP as a model system to study presenilin [9]. However, SPP has significant differences with presenilin. For example, SPP appears to have a membrane topology opposite to that of presenilin [20], a characteristic that could be related to the fact that presenilin cleaves type I membrane proteins whereas SPP cleaves type II ones. In addition, contrary to presenilin, SPP does not require complexation with additional proteins for activity.

The above mentioned bioinformatics study by Ponting *et al.* also raised the hypothesis that archaea might contain presenilin-like proteins [19]. To date however, archaeal GXGD-type diaspartyl intramembrane proteases with biochemical similarities to presenilins or SPPs have not been reported. Here, we screened twelve commercially available archaeal genomes for the presence of putative intramembrane proteases harboring the YD and GXGD presenilin and SPP signature motifs within adjacent TMDs. Expression and purification trials of cloned targets identified the protein MCMJR1 from the euryarchaeon *Methanoculleus marisnigri* JR1, as an optimal expressor that was stable during purification in detergent. Using *in vitro* proteolytic assays, site-directed mutagenesis experiments and cleavage site determination by mass spectrometry we demonstrated that MCMJR1 is indeed an archaeal GXGD-type intramembrane protease with significant biochemical similarities compared to presenilins and the SPPs.

## Results

### Identification of putative GXGD-type diaspartyl intramembrane proteases from archaea

Despite limited areas of direct sequence homology [19], presenilins and SPPs share a multispanning membrane topology and identical YD and GXGD signature motifs carrying the two catalytic aspartates on adjacent TMDs, as well as a PAL motif near their C-termini (Fig. 1A). We used these signature motifs as seeds to identify putative GXGD-type diaspartyl intramembrane proteases among a total of 12 commercially available genomes from archaea (Results S1). We selected and cloned 12 targets as fusion proteins containing His_10_-tags at either the N- or C-termini. We expressed these in small scale in *E. coli* and purified them by Ni-NTA chromatography in either Fos-choline 12 (FC-12) or dodecyl-β-D-maltopyranoside (DDM), two non-denaturing detergents commonly used as efficient solubilizers of bacterial inner membranes (Fig. 1B). One candidate (we termed it MCMJR1), from *Methanoculleus marisnigri* strain *JR1*, could be expressed and purified in much greater yields relative to all the other targets. For scaled-up production, MCMJR1 was expressed in *E. coli* carrying a N-terminal SUMO-tag. This strategy yielded milligram quantities (Fig. 1C) of highly pure protein (Fig. 1D) for *in vitro* functional characterization. Analysis of the MCMJR1 sequence using several membrane protein topology prediction softwares postulated a multispanning membrane topology with eight predicted TMDs. The signature YD and GXGD catalytic motifs of presenilin and SPP were predicted to be in adjacent TMDs 5 and 6, which were joined by a long loop.

### MCMJR1 is a GXGD-type diaspartyl intramembrane protease

**Figure 2:**
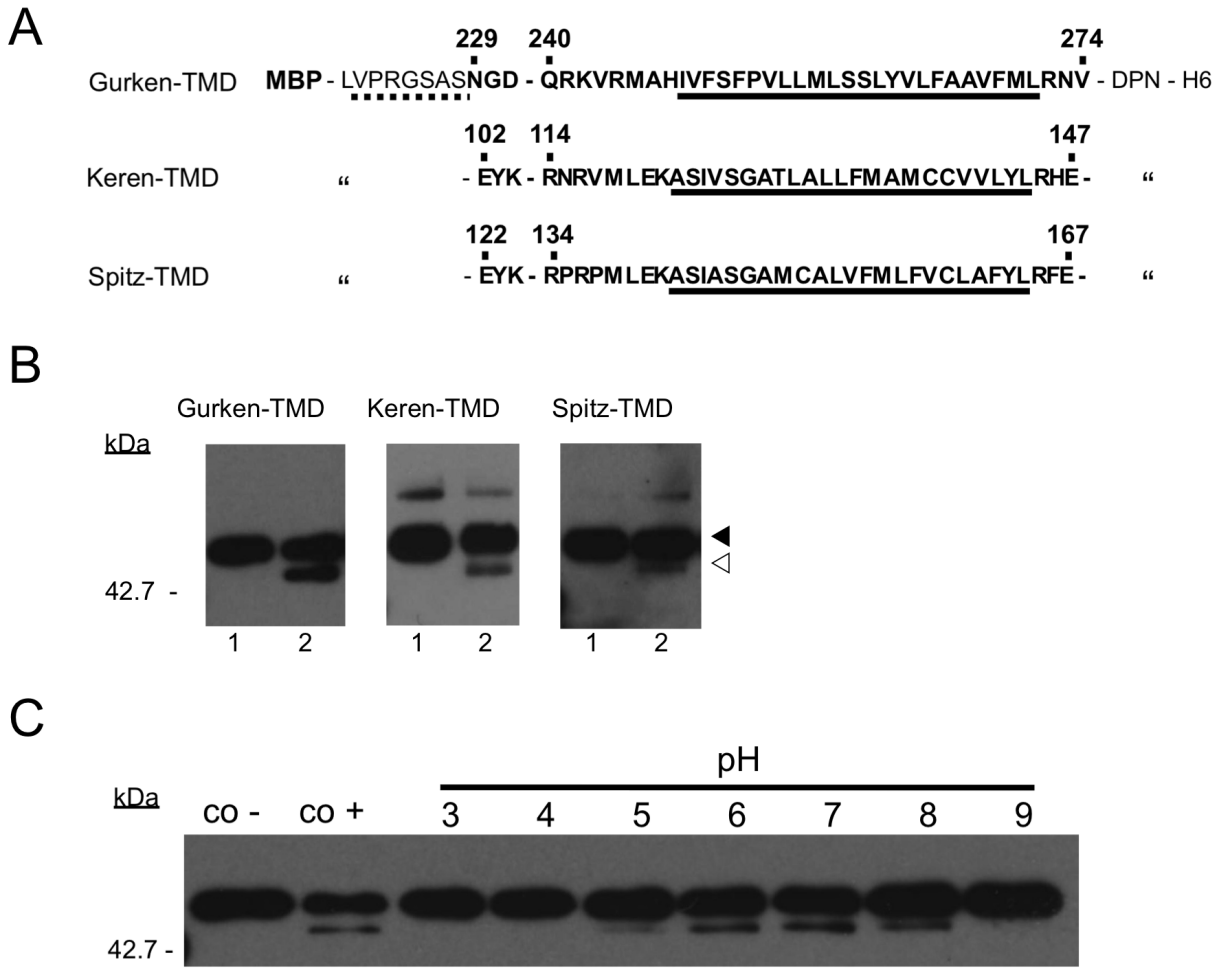
Proteolytic activity of MCMJR1. **A.** Sequence detail of the chimeric substrates Gurken-TMD, Keren-TMD and Spitz-TMD. A thrombin cleavage site (discontinuous underline) separates MBP from the amino acid sequence corresponding to the natural substrate (bold) and the C-terminus includes a His_6_-tag (H6) for purification. The predicted TMD is underlined. Specific amino acid residues are numbered according to the wild-type protein. **B.** An anti-MBP western blot analysis of Gurken-TMD, Keren-TMD and Spitz-TMD after incubation in 0.1% DDM at 37°C for 8 hours in the absence (lane 1) and presence of MCMJR1 (lane 2). Protein bands corresponding to the undigested (black arrowhead) and digested (white arrowhead) substrates are indicated on the right and the molecular weight marker position is shown on the left side. **C.** An anti-MBP western blot analysis of the pH dependence of MCMJR1 activity. MCMJR1 purified in 5 mM NaHepes at pH 7.0 was diluted 8-fold (to a final concentration of 0.5 µM) into a solution containing Gurken-TMD (0.5 µM) in either a 50 mM Bicine buffer at pH 9.0, 50 mM NaHepes buffer at pH 8.0–7.0, 50 mM Bis-Tris buffer at pH 6.0, and 50 mM Sodium Acetate-Acetic acid buffer at pH 5.0–3.0. As negative and positive controls Gurken-TMD was incubated at pH 7.0 in the absence (co −) and presence (co +) of MCMJR1.

To determine if MCMJR1 displays protease activity we used an *in vitro* assay. *In vitro* cell-free assays using detergent-solubilized components constitute reliable tools to monitor intramembrane protease activity. For example, *in vitro* γ-secretase activity is measured routinely using a recombinant substrate derived from APP [23]. In another example, the activity of SPP [22] has been probed by incubating the purified protease with chimeric proteins based on physiological substrates and following their degradation by western blotting and SDS-PAGE. Here we used a comparable approach, originally developed in our laboratory to assay the activity of rhomboid serine intramembrane proteases. The chimeric substrates are genetically engineered as fusion proteins between bacterial maltose binding protein (MBP) and the TMDs of physiological *Drosophila* rhomboid-1 substrates Gurken, Keren and Spitz (Fig. 2A). In this assay, purified substrate and enzyme are incubated in a detergent-containing buffer at 37°C for a defined length of time, typically 8 hours or overnight. Intramembrane protease activity produces a ∼42 kDa MBP fragment which can be identified either by direct staining of SDS-PAGE gels or by blotting using anti-MBP antibodies, and efficiently distinguished from the undigested ∼50 kDa substrate. Incubation of MCMJR1 with the substrates Gurken-TMD and Keren-TMD, in the presence of amino-peptidase (bestatin) and cysteine/serine protease (E-64/PMSF) inhibitors, produced a ∼42 kDa band representative of the MBP moiety, as judged by anti-MBP western blotting (Fig. 2B). The TMD of Gurken appears to be the most efficiently cleaved substrate. Moreover, although Spitz-TMD and Keren-TMD display high sequence homology, Spitz-TMD was poorly processed compared to Keren-TMD. The pH dependence of MCMJR1 activity (Fig. 2C) indicated a pH optimum of ∼7.0, which is similar to the pH optimum displayed by detergent solubilized γ-secretase [23] and SPP [24].

**Figure 3:**
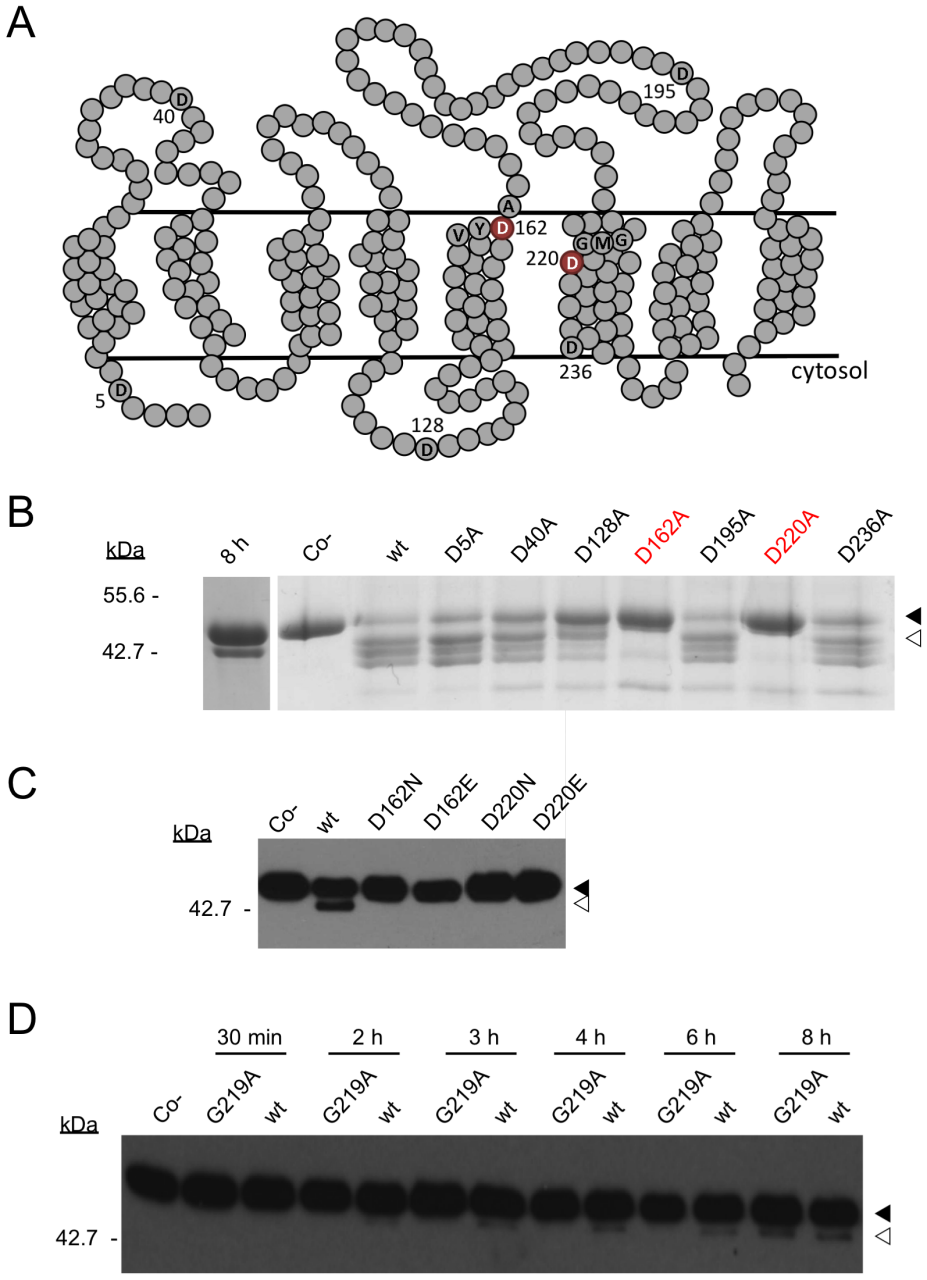
Identification of catalytic aspartic acid residues in MCMJR1. **A.** A prediction of the membrane-spanning regions and their orientation was obtained from the amino acid sequence of MCMJR1 using three independent topology prediction softwares TMpred, Toppred, and HMMTOP. Horizontal lines delimit the lipid bilayer. All the aspartic acid residues (D5, D40, D128, D162, D195, D220 and D236) in MCMJR1 are shown. Amino acids flanking the candidate catalytic aspartic acid residues (in red) are also depicted. **B**. A coomassie stained 10% SDS-PAGE analysis of a 32-hour incubation of Gurken-TMD in the absence of MCMJR1 (co −) and in the presence of wild-type MCMJR1 (wt) and aspartate-to-alanine single mutants. Digestion products (white arrowhead) could be detected for wild-type MCMJR1, as well as D5A, D40A, D128A, D195A and D236A mutants. In contrast, the D162A and D220A mutants (in red) showed no activity. A 8-hour incubation with wild-type MCMJR1 (8 h) is also included to show that the additional product bands observed after a 32-hour incubation are the result of an extended reaction time. **C**. An anti-MBP western blot analysis of the incubation of Gurken-TMD in the absence of MCMJR1 (Co−) and in the presence of wild-type MCMJR1 (wt), and D162N, D162E, D220N and D220E mutants. Digestion products (white arrowhead) could be detected for wild-type MCMJR1 but not for the mutants. **D.** Slowed rate of intramembrane proteolysis by the G219A MCMJR1 mutant. An anti-MBP western blot analysis of Gurken-TMD following incubation with wild-type (wt) MCMJR1 and the mutant G219A. Aliquots of the digestion reaction were analyzed after 30 min, 2, 3, 4, 6 and 8 hours. Co − denotes Gurken-TMD in the absence of MCMJR1. Protein bands corresponding to the undigested (black arrowhead) and digested (white arrowhead) substrate Gurken-TMD are indicated and the molecular weight marker positions are shown on the left.

To confirm the observed proteolytic activity was MCMJR1-specific and to probe the role of aspartic acid residues in the function of this protein, we carried out a site-directed mutagenesis study. In addition to D162 and D220 located in the YD and GXGD motifs respectively, there are another five aspartic acid residues in MCMJR1 (D5, D40, D128, D195 and D236; Fig. 3A). Each one was mutated into alanine and the resulting single amino acid variants were expressed, purified and tested for activity against chimeric substrates (Fig. 3B). The yield and purity of each Asp-to-Ala MCMJR1 mutant was comparable to that obtained for the wild-type protein. Mutation of either D162 or D220 to alanine completely abolished enzymatic activity. In contrast, the five other Asp-to-Ala mutants had no apparent effect on the proteolytic function of MCMJR1. We note that under the 32-hour incubation time employed to ascertain the lack of activity of the catalytic mutants, two additional bands appear around the region where the product migrates. These two bands are not apparent under our typical 8-hour incubation (Fig. 3B; 8 h) and they do not react with anti-MBP (Fig. 3C), and thus we believe they might constitute unspecific cleavage products. In the event that the effect observed for the Asp-to-Ala mutations of D162 and D220 was structural rather than functional, we introduced an isosteric asparagine and a glutamic acid to conserve the negative charge. These mutations abolished MCMJR1 activity (Fig. 3C), demonstrating that aspartates are specifically required at these two positions. All together, these data demonstrate that the observed protease activity is indeed MCMJR1 specific, and offer strong support to the notion that the enzyme is a GXGD-type diaspartyl protease harboring its two catalytic residues in the same YD and GXGD amino acid motifs that are the signatures for presenilin and SPP.

To further evaluate the role of the GXGD motif in MCMJR1, we mutated the glycine immediately preceding the second catalytic aspartate to alanine (G219A). In presenilin, the corresponding mutant (G384A) significantly decreases the rate of production of Aβ40 [25] and a similar effect was also reported in the SPP homolog SPPL2b [26]. The G219A mutant (GXG_219_D) in MCMJR1 also decreased its rate of proteolysis (Fig. 3D), suggesting that the role of this residue adjacent to the catalytic D220 might be similar to that reported for SPP and presenilin.

### MCMJR1 cleaves at multiple sites within the hydrophobic TMD of the substrate

**Figure 4:**
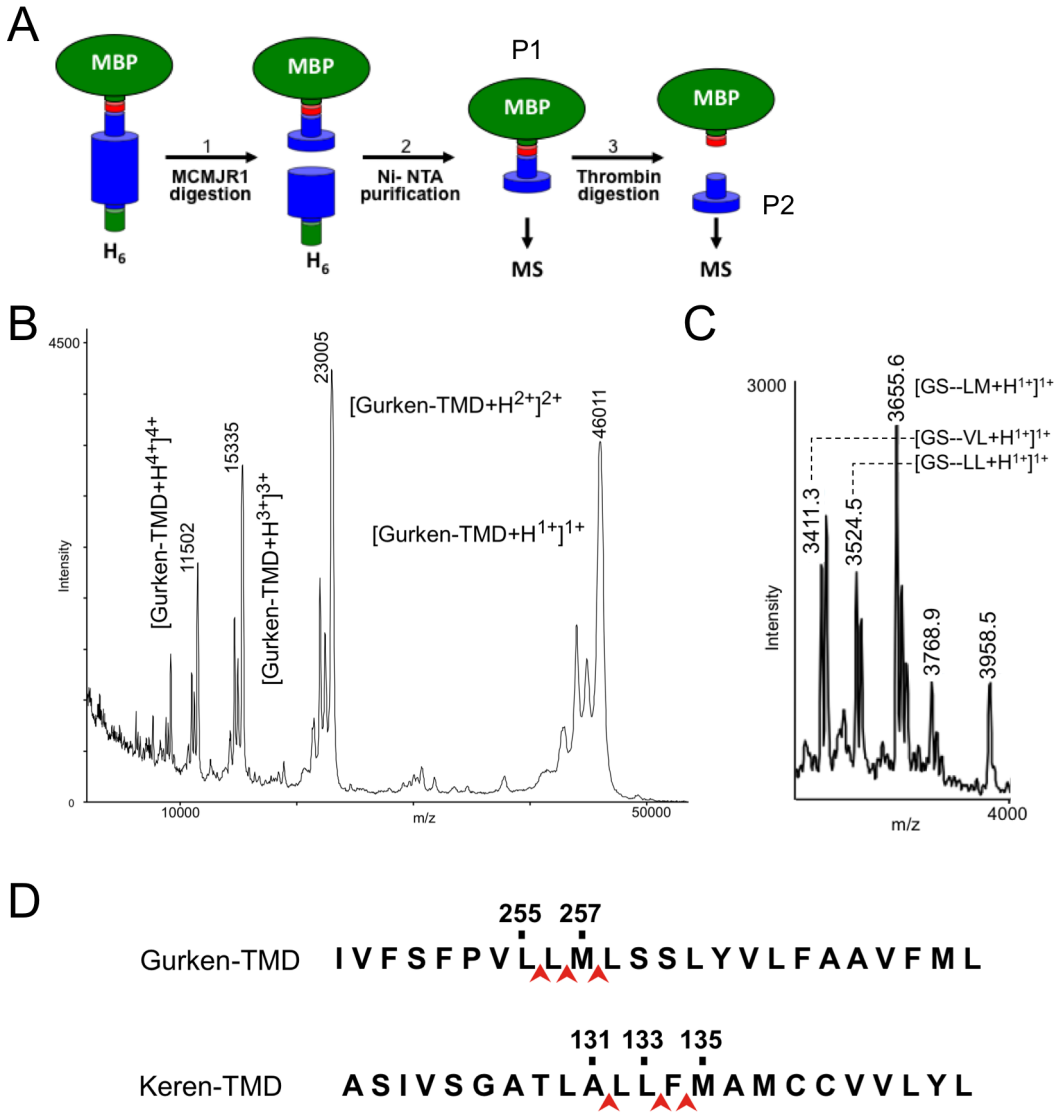
MCMJR1 cleaves within the TMD of the substrates. **A.** Schematic of the processing of MBP-based chimeric substrates by MCMJR1 and generation of samples for mass spectrometry analysis. The wider cylinder represents the TMD region and the engineered thrombin site (LVPR/GS) is colored in red. P1 denotes the N-terminal MBP containing product of the digestion of the substrate chimera by MCMJR1. P2 denotes the product of the digestion of P1 by thrombin. **B.** Mass spectrum recorded in positive ionization mode of the N-terminal product (corresponding to P1 in **A**) after digestion of Gurken-TMD by MCMJR1. The observed *m/z* for selected peaks is given. Single, double, triple and quadruple charged species are also indicated. **C.** Mass spectrum recorded in positive ionization mode of the peptide (corresponding to P2 in **A**) between the thrombin cleavage site and the MCMJR1 cleavage site in Gurken-TMD. **D.** Schematic representation of the proteolytic profile of MCMJR1 against the substrates Gurken-TMD and Keren-TMD.

Next, we addressed the question of whether MCMJR1 cleaves substrates within their TMD. To determine the proteolytic profile of MCMJR1 we used matrix-assisted laser desorption/ionization time-of-flight (MALDI-TOF) mass spectrometry. Cleavage of the chimeric substrates by MCMJR1 generates an N- and C-terminal product. The C-terminal product contains the majority of the TMD and thus will bind substantial amounts of detergent, which can hamper mass spectrometry analysis [27]. We therefore focused our analysis on the N-terminal product, the bulk of which is constituted by MBP followed by a hydrophilic C-terminal linker. As an example, figures 4A–C show the cleavage site determination procedure for MCMJR1 in its reaction with Gurken-TMD. Following incubation, using a concentration of substrate and enzyme that maximizes the yield of the reaction, an efficient degradation of the substrate could be achieved. Ni-NTA resin was added to this reaction mixture to sequester the uncleaved substrate and the C-terminal fragment (both containing His_6_-tags), and thus yield a purified N-terminal product. TCA precipitation of this species followed by MALDI-TOF mass spectrometry analysis (Fig. 4B) yielded a mass spectrum showing four main peaks at *m/z* values, corresponding to single, double, triple and quadruple protonated species with mass of ∼46011 Da. Comparison of the calculated masses of possible N-terminal reaction products suggests that MCMJR1 cleaves Gurken-TMD after Leu256 (Table 1). To verify the location of the cleavage, the N-terminal species derived from the digestion of Gurken-TMD was incubated with thrombin. Thrombin treatment of this product produces a small peptide spanning from the engineered thrombin site to the MCMJR1 cleavage site (Fig. 4A), which can be measured with increased accuracy due to its relatively small mass. The mass spectrometry analysis of this product of double digestion (Fig. 4C) confirmed the identity of the cleavage site after Leu256 and identified two additional sites after Leu255 and Met257 (Table 1). An identical procedure was employed to analyze the cleavage of Keren-TMD by MCMJR1. In this case, mass spectrometry analysis again revealed one main cleavage site after Leu133 and two additional secondary sites after Leu131 and Phe134 (Table 1; Fig. 4D). These data suggest that MCMJR1 cleaves within the TMD region of the substrate, with the existence of secondary cleavage sites.

**Table 1:** Observed and calculated masses of MCMJR1 cleavage products.

Substrate	Product sequence	Calculated mass of products (Da)	Observed mass of products (Da)
Gurken-TMD	**MBP**-PRGS–PVLL_256_	46014.1	46011
	GS–PVL_255_	3409.9	3411.3
	GS–PVLL_256_	3523.1	3524.5
	GS–PVLLM_257_	3654.3	3655.6
Keren-TMD	**MBP**-PRGS–TLALL_133_	46332.4	46338
	GS–TLA_131_	3615.1	3615.8
	GS–TLALL_133_	3841.4	3842.2
	GS–TLALLF_134_	3988.6	3989.5
APP-TMD	**MBP**-PRGS–VGG_709_	43680.4	43674
	**MBP**-PRGS–VGGVV_711_	43878.7	43860

### Inhibition of MCMJR1 by an established presenilin transition state analog inhibitor

**Figure 5:**
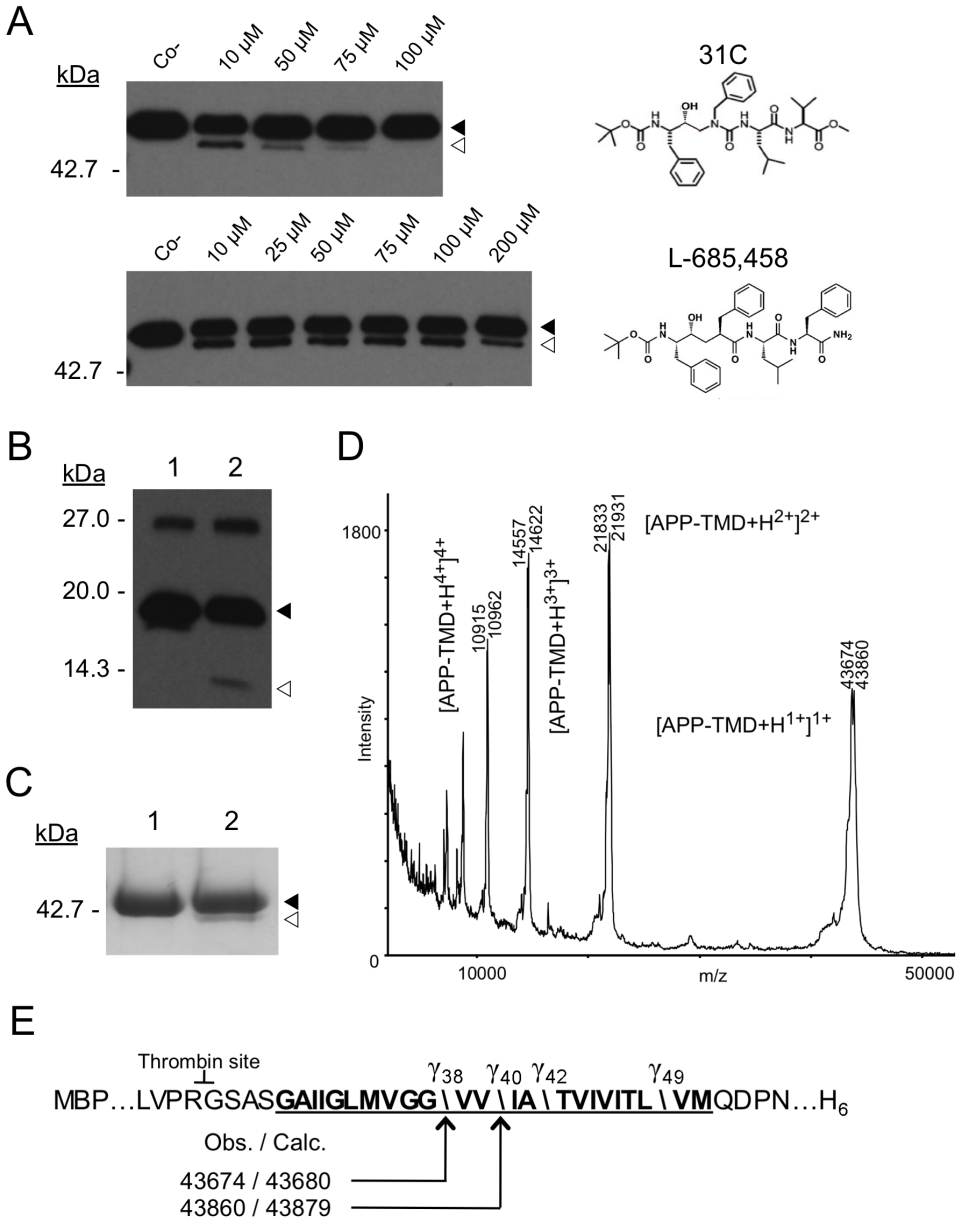
MCMJR1 can cleave γ-secretase substrates derived from APP. **A.** An anti-MBP western blot analysis of MCMJR1 inhibition. The substrate Gurken-TMD was incubated with MCMJR1 in the presence of increasing amounts of the γ-secretase inhibitors 31C (upper panel) and L685,458 (lower panel). Co− denotes Gurken-TMD in the absence of MCMJR1. The chemical structures of these inhibitors are depicted on the right. **B.** Anti-Flag immunoblot analysis of C100Flag in the absence (lane 1) and presence of MCMJR1 (lane 2). Proteolysis by MCMJR1 is indicated by the presence of a band that cross-reacts at a lower molecular weight (white arrowhead) compared to the intact substrate (black arrowhead). A band corresponding to C100Flag dimer also cross-reacts at ∼27 kDa. **C.** A coomassie stained 10% SDS-PAGE analysis of APP-TMD alone (lane 1) and after incubation with MCMJR1 (lane 2). The full-length substrate (black arrowhead) and N-terminal product (white arrowhead) generated after cleavage by MCMJR1 are marked. **D.** Mass spectrum recorded in positive ionization mode of the N-terminal product after digestion of APP-TMD by MCMJR1. The observed *m/z* for selected peaks is given. Single, double, triple and quadruple charged species are also indicated. **E.** Schematic showing the proteolytic profile of MCMJR1 on APP-TMD (black arrows) along with the calculated (calc) and observed (obs) mass. The region corresponding to the TMD of APP is underlined. The thrombin cleavage site following MBP is also shown. The known cleavage sites of γ-secretase to generate Aβ at positions 38, 40, 42 and 49 are marked (backslash) for comparison.

SPP and presenilin share pharmacological similarities as they are targeted by many of the same small molecules, including transition state analogs, non-transition state inhibitors, and modulators [28]. Moreover, both enzymes apparently have a substrate-binding site that is distinct from the active site and certain nonsteroidal anti-inflammatory drugs known to shift the site of proteolysis by γ-secretase also affect SPP [22]. Here we asked the question of whether established inhibitors of presenilin and SPP could also have an effect on MCMJR1. Figure 5A shows inhibition of MCMJR1 activity against Gurken-TMD by 31C, a transition state analog inhibitor known to target presenilin [29] and SPP [22]. Complete inhibition of activity was achieved with ∼100 µM 31C, as judged by densitometry. These data further reinforce the notion that MCMJR1 displays relevant biochemical similarities to presenilin and SPP. Remarkably however, L-685,458 [15], another established presenilin and SPP inhibitor that is structurally similar to 31C, failed to impact MCMJR1 activity under the same assay conditions.

### MCMJR1 displays proteolytic activity against substrates derived from the amyloid precursor protein

Given the biochemical similarities between MCMJR1 and presenilin we questioned whether MCMJR1 could cleave *bona fide* presenilin substrates, unique to this enzyme. γ-Secretase cleaves at multiple sites within the TMD of its physiological substrate βCTF, derived from the processing of the APP by BACE [30]. A recombinant form of βCTF, known as C100Flag and originally designed by Li *et al* [23], has been used extensively to assay γ-secretase activity both *in vitro* and *in vivo* [23], [29], [31]. Incubation of C100Flag with MCMJR1 resulted in the appearance of an anti-Flag immunoreactive band at ∼8 kDa, which was consistent with the expected molecular weight of intramembrane proteolysis products (Fig. 5B). To map the profile of this cleavage, we designed a chimeric substrate (APP-TMD) containing the TMD of APP fused to MBP. Incubation of MCMJR1 with APP-TMD resulted in the appearance of a ∼42 kDa band corresponding to the MBP moiety of the chimeric substrate (Fig. 5C). The product of this reaction was purified and analyzed by MALDI-TOF mass spectrometry (Fig. 5D). Two cleavage sites within the TMD of APP were observed (Table 1). Remarkably, these sites corresponded to the Aβ38 and Aβ40 γ-secretase cleavage sites (Fig. 5E).

## Discussion

**Figure 6:**
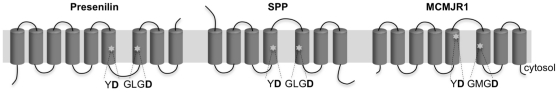
Topologies of presenilin, SPP and MCMJR1. A schematic of the predicted topologies of presenilin [10], SPP [20] and MCMJR1. Cylinders across the lipid bilayer (a continuous slab) depict the predicted TMD regions. Stars mark the positions of the catalytic aspartic acid residues.

In this report we introduce MCMJR1 as a novel GXGD-type diaspartyl intramembrane protease from archaea. A key question is how does MCMJR1 compare to the eukaryotic GXGD-type diaspartyl intramembrane proteases, presenilin and SPP. The overall sequence homology between presenilins and SPPs is very low, except for the fact that they both share a common architecture with multiple TMDs (Fig. 6), an absolute conservation of the signature YD and GXGD catalytic motifs present in adjacent TMDs and a C-terminal PAL motif [19]. Remarkably, despite low sequence homology with the eukaryotic enzymes, there appears to be a conservation of these signature motifs and topological features in MCMJR1. Indeed, mutation to alanine of any of the aspartic amino acid residues within the sequences YD_162_ or GXGD_220_ abolishes MCMJR1 proteolytic activity, as does the corresponding mutations in presenilins [11] and SPPs [20]. In addition, the mutation G219A - adjacent to D220 in the GXGD motif - slows down proteolytic activity considerably, in clear analogy to the effect observed when the corresponding mutation was introduced in both presenilin [25] and SPP [26]. The apparent reason for the observed reduction in activity of the Gly-to-Ala mutant in presenilin is that this glycine is key to allow rotational freedom of the adjacent catalytic aspartate [25]. The role of this residue in MCMJR1 might be comparable to that reported for presenilins. Thus, we suggest that the function of the signature YD and GXGD motifs in presenilins and SPPs is conserved also in MCMJR1. Although we cannot at this point be fully confident about the validity of the predicted topology of MCMJR1 (Fig. 6), we note that in analogy to presenilins and SPPs these two motifs are located towards the C-terminal of MCMJR1, in adjacent TMDs that are joined by a relatively long loop. The proteolytic activity of presenilin is dependent on the loop between the two TMDs that harbor the catalytic motifs undergoing endoproteolysis to yield N- (NTF) and C-terminal (CTF) fragments of the enzyme [32]. This endoproteolytic event in presenilin is thought to cut and remove a helix that otherwise obstructs the active site in the immature enzyme [33]. In analogy to SPP [20], MCMJR1 does not appear to depend on endoproteolysis for catalysis. We note however that the integrity of this loop appears to be critical for MCMJR1 activity, as multiple loop truncation mutants were found to be inactive in our *in vitro* assay (Fig. S1). As a salient difference, MCMJR1 does not contain an obvious C-terminal PAL motif. Biochemical studies with presenilin and SPP have suggested this motif to be functionally important and to contribute to the architecture of the active site [34]. Instead, a tantalizing PPL sequence is present just before the last TMD of MCMJR1. The functional role of this sequence in MCMJR1, if any, remains to be determined.

From a biochemical perspective significant similarities and differences between MCMJR1 and presenilins and SPPs are also evident. To date, presenilin has been found responsible for the intramembrane cleavage of a growing list of type I integral membrane proteins, which appear not to share any consensus sequence around their transmembrane cleavage sites [35] and the main prerequisite for proteolysis seems to be prior enzymatic removal of an ectodomain [36]. In contrast, substrate ectodomain shedding appears unlikely to be a pre-requisite for SPP-mediated intramembrane proteolysis, although there is evidence that it may facilitate cleavage [37], [38], [39]. In analogy to SPP, ectodomain removal appears not to be a requisite for MCMJR1-catalyzed proteolysis. In further analogy to SPPs, MCMJR1 appears to be active in the absence of accessory proteins. Remarkably, MCMJR1 displays a certain degree of specificity towards the TMD region of the substrates. Indeed, Keren-TMD could be more efficiently hydrolyzed than Spitz-TMD, despite the two substrates having comparable TMD regions. A salient feature of presenilin is its ability to cleave substrates at multiple sites within their TMD [4]. In contrast, SPP-catalyzed intramembrane proteolysis seems to occur predominantly at one position after a helix-breaking amino acid residue [22]. Our mass spectrometry data suggest that in analogy to presenilin, MCMJR1 displays promiscuous peptide bond specificity as processing of the chimeric substrates was shown to occur at multiple positions within the hydrophobic region of the predicted TMD. Perhaps the single most remarkable feature distinguishing MCMJR1 from SPP, is its unique ability to hydrolyze substrates derived from APP. Indeed, MCMJR1 was able to cleave the established *in vitro* presenilin substrate C100Flag [23]. Moreover, the chimera containing the TMD of APP was hydrolyzed at two transmembrane sites, which correspond to known presenilin cleavage positions. To the best of our knowledge the ability of SPP to cleave APP-derived substrates has not been reported. This feature of MCMJR1 deserves further investigation with enzyme and substrates reconstituted in lipid bilayers to best mimic an *in vivo* situation. However, it is remarkable that an enzyme from archaea can produce Aβ40, and do so without the apparent need of the co-factors APH-1, nicastrin, and PEN-2 that are required for γ-secretase function.

The discussed features distinguish MCMJR1 from prokaryotic type 4 prepilin peptidases (TFPP; [40]), which are also GXGD-type diaspartyl proteases, but contain their catalytic aspartates within soluble regions that are separated by several TMDs and cleave their substrates outside the membrane [40], [41]. Sequence database searches suggest that presenilin and SPPs are well conserved throughout eukaryotes with putative homologues present in yeast, plants, mollusks, insects, fish, birds and mammals [19]. The very limited sequence homology suggests that these two families might have evolved by convergence and are probably not directly related. The existence of presenilin-like proteins in archaea had been suggested [19], but never proven biochemically. The demonstration that archaeal MCMJR1 is a GXGD-type diaspartyl intramembrane protease with biochemical similarities to presenilin and SPP suggests that this type of proteolytic activity might have an older origin than previously anticipated.

Presenilin is currently the subject of intense investigation due to its relevance to human biology and to the pathogenesis of AD [8]. However, as presenilin is active only when in complex with APH-1, nicastrin, and PEN-2 [16], and also has to undergo endoproteolytic activation [32], the pace in our mechanistic and structural understanding of this enzyme has been understandingly slow. The discovery of human SPP has contributed significantly to our knowledge of this type of proteases in general, and on presenilin in particular [9]. Still, the lack of more tractable and presumably simpler GXGD-type diaspartyl intramembrane proteases from prokaryotic sources has hampered the implementation of adequate biophysical and structural approaches to study this important class of membrane enzymes. Indeed, the availability of prokaryotic homologs has resulted in structural and functional breakthroughs in the related field of rhomboid [42], [43], [44], [45] and S2P [46] intramembrane proteases. Despite the fact that the physiological substrates of MCMJR1 are hitherto unknown, its ability to recapitulate key biochemical properties of eukaryotic presenilins and SPPs make this archaeal enzyme an optimal system for high-resolution structure determination and in depth studies on the mechanism of GXGD-type diaspartyl intramembrane proteases.

## Materials and Methods

### Identification and expression trials of putative GXGD-type diaspartyl intramembrane protease genes from archaeal genomes

A simple genomic expansion was performed by BLAST based on published sequences of putative GXGD-type diaspartyl intramembrane proteases [19]. The search was confined to commercially available archaeal genomes. Short (21 bp) oligonucleotides were designed to match the 5′ and 3′ ends of the coding regions, and the target genes amplified by PCR using standard procedures, cloned into a shuttle vector (pGEM-T easy system, Promega, Inc.) and sequenced from both directions to confirm their identity before being re-cloned into appropriately designed expression vectors.

Target archaeal GXGD-type diaspartyl intramembrane protease genes were cloned into two variants of T7 promoter-based and isopropyl-β-D-thio-galactoside (IPTG) inducible pET19a (Novagen, Inc.) vectors for expression as either N or C terminal His_10_-tag fusion proteins. For subsequent scale-up experiments the MCMJR1 gene was transferred to a variant of pET-28b for expression of a genetically engineered N-terminal fusion with the small ubiquitin modifying protein (SUMO; [47]), preceded by a His_6_-tag. All expression experiments were performed in *E. coli* strain Rosetta (DE3) pLysS (Novagen, Inc.). Transformed cells were grown to mid-log phase at 37°C in 2xTY medium before lowering the temperature to 18°C and inducing protein expression with the addition of IPTG to a final concentration of 0.1 mM. Protein expression was allowed to continue for 18 hours at 18°C. Initial expression tests were performed on a 100 mL scale. Scale-up of protein production was instead carried out on 800 mL scale, using 2 L baffled flasks as vessels (Bellco Glass, Inc.). Cells were harvested by centrifugation at 6,000× g. Single aspartate MCMJR1 mutants were generated using the Quickchange™ (Stratagene, Inc.) site-directed mutagenesis kit, following protocols provided by the manufacturer. Expression and purification of these mutants was carried out essentially as for the wild-type protein.

A model for the topology of MCMJR1 was obtained by analyzing its sequence using three independent topology prediction softwares TMpred (http://www.ch.embnet.org/software/TMPRED_form.html), Toppred, (http://mobyle.pasteur.fr/cgi-bin/portal.py?form=toppred) and HMMTOP (http://www.enzim.hu/hmmtop/html/submit.html). We selected the N-terminus to be cytosolic, because the N-terminal SUMO-tag used for expression in *E. coli* does not have a signal sequence and it is cytosolic.

### Expression of chimeric substrates in *E. coli*

The chimeric protein substrates used for the *in vitro* activity assay contained the predicted TMD region of the 695-amino acid long isoform of APP (βAPP695; GeneBank accession number CAA68374; TMD region Gly625-Met647) and that of EGFR ligands of *Drosophila melanogaster*, namely Spitz (GeneBank accession number NP_476909; TMD region Ala142-Leu164), Keren (GeneBank accession number NP_524129; TMD region Ala122-Leu144) and Gurken (GeneBank accession number NP_476568; TMD region Ile248-Leu271). The cDNAs for Gurken, Keren and Spitz were obtained from the Drosophila Genomics Resource Center and MBP (without a signal sequence) was amplified from plasmid H-MBP-3C [48]. The TMD region of APP695 was cloned from a synthetic duplex DNA fragment. The chimeras also included a unique thrombin cleavage site (LVPR/GS) to aid in the ensuing mass spectrometry experiments and a C-terminal His_6_-tag for purification. These chimeras termed Gurken-TMD, Keren-TMD, Spitz-TMD and APP-TMD were subcloned into pET-29b plasmids for expression in *E. coli* strain BL21 (DE3). Colonies were picked and grown to mid-log phase at 37°C in 1 L of growth medium (0.85% Na_2_HPO_4_, 0.03% KH_2_PO_4_, 0.5 g/L NaCl, 0.01% NH_4_Cl, 10 g/L Tryptone, 5 g/L Yeast Extract, 2 mM MgSO_4_.7H_2_O, 1 mM CaCl_2_, 0.6% Glucose, 10 µg/mL Thiamine) supplemented with 40 µg/mL Kanamycin. Cell growth was allowed to continue for 4 hours at 37°C following induction of protein expression with 0.4 mM IPTG.

The pET21 expression vector (Novagen, Inc.) carrying the recombinant C100Flag [23] substrate (108 amino acid residues; MW = 12.3 KDa), which corresponds to the C-terminal fragment (CTF; residues Met596-Asn695) derived from APP695 (GeneBank accession number CAA68374) with an extra C-terminal Flag-tag, was a kind gift from Dr. Yueming Li (Sloan-Kettering, New York). This plasmid was used to transform *E. coli* host strain BL21 (DE3). The cell culture was grown at 37°C to an OD at 600 nm of ∼0.7 before protein expression was induced by addition of IPTG to 0.4 mM final concentration. Following induction, cell growth was allowed to continue for 4 hours at 37°C.

### Protein purification

All procedures were performed on ice or at 4°C. Cells were resuspended in buffer A (20 mM NaHepes pH 7.5, 250 mM NaCl, 1 mM MgSO_4_, 1 mM β-mercaptoethanol containing 0.1 µg/mL DNAse, 4 µg/mL of E-64 (Alexis biochemicals, Inc.), 14 µg/mL of Bestatin (Alexis biochemicals, Inc.) and 100 µM of PMSF (Sigma-Aldrich). Cells were then lysed in a 35 mL French pressure cell operating at 11,000 psi. Cell debris was cleared by centrifugation at 10,000× g for one hour. Cytoplasmic membranes were isolated by centrifugation at 100,000× g for 1 hour, resuspended at ∼5 mg/mL total protein concentration in buffer A and solubilized with 1% (w/v) detergent. Alternatively, the crude lysate was solubilized directly by addition of 1% (w/v) detergent at a ratio of 10∶1 (wet cell mass to detergent). Solubilization was performed at 4°C under gentle rotation for one hour with either DDM (Anatrace, Inc) or FC-12 (Anatrace, Inc.). Insoluble material was removed by ultracentrifugation at 148,000× g for 1 hour and the supernatant containing the detergent-solubilized membranes was supplemented with imidazole buffered at pH 7.0 to a final concentration of 50 mM and added to pre-equilibrated Ni-NTA resin (Qiagen) at a ratio of 1∶50 (resin to solution, v/v). After 2 hours of incubation under gentle rotation, the affinity resin was poured onto a disposable column and washed with 4 column volumes of buffer B (20 mM Na-Hepes pH 7.5, 250 mM NaCl, 1 mM β-mercaptoethanol) supplemented with 50 mM imidazole and either 0.1% DDM or 0.125% FC-12. Histidine-tagged proteins were eluted in buffer B supplemented with 250 mM imidazole and either 0.1% DDM or 0.125% FC-12. For SUMO fusion constructs, the samples were treated overnight with ULP1 (added at ∼1∶20 protease to substrate ratio, w/w) to allow the proteolytic release of the SUMO-tag while dialyzing against a 20 mM Na-Hepes pH 7.0 buffer containing 250 mM NaCl, 2 mM β-mercaptoethanol and either 0.05% DDM or 0.0625% FC-12. After re-passing the mixture through pre-equilibrated Ni-NTA resin, histidine-tagged ULP1 protease, SUMO tag and any impurities were retained, while the proteins of interest were collected in the flow-through. Finally, an aliquot from the this sample was use for *in vitro* activity assays or, to increase purity even further, the entire preparation was concentrated to under 500 µL before loading onto a superdex 200 HR10/30 (GE Healthcare, Inc) size-exclusion chromatography column equilibrated in buffer B containing either 0.05% DDM or 0.08% FC-12.

### Substrate production

Cells expressing MBP chimeric substrates were collected by centrifugation and resuspended in 15 mL of buffer C (50 mM Tris-HCl pH 7.4, 300 mM NaCl, 10 mM Imidazole, 10% glycerol) containing 0.1 µg/mL DNAse, 4 µg/mL of E-64, 14 µg/mL of Bestatin and 100 µM of PMSF. Cells were then lysed in a 35 mL French pressure cell operating at 11,000 psi., and the intact cells and cellular debris spun down at 43,000× g for 15 minutes. Bacterial membranes were isolated by ultracentrifugation at 100,000× g for 45 minutes and solubilized in a total volume of 25 mL of buffer C containing 2% Triton X-100 (Anatrace, Inc.; TX-100) for 4 hours. The insoluble material was separated by ultracentrifugation at 100,000× g for 45 minutes and the supernatant incubated with 0.5 mL of Ni-NTA resin pre-equilibrated in buffer C. After 4 hours at 4°C, the resin was packed onto a disposable column and the detergent exchanged to 0.1% DDM by washing the resin with buffer C containing 0.2% TX-100 first, then with buffer C containing 0.1% DDM and finally with 3 mL of buffer D (50 mM Tris-HCl pH 7.4, 300 mM NaCl, 20 mM Imidazole, 10% glycerol) containing 0.1% DDM. The substrate was eluted with buffer E (50 mM Tris-HCl pH 7.4, 300 mM NaCl, 250 mM Imidazole, 10% glycerol) with 0.1% DDM. Finally, the protein-containing solution was dialyzed against phosphate buffered saline (PBS) at pH 7.4 with the addition of 10% glycerol and 0.1% DDM.

Bacterial membranes containing C100Flag were isolated and solubilized as described above. The insoluble material was separated by ultracentrifugation and the supernatant incubated with 300 µL of pre-equilibrated anti-flag M2 affinity resin (Sigma, Inc.). After 1 hour of slow rotation at 4°C, the M2 resin was first washed with 3×5 mL PBS containing 0.2% TX-100 and then with 3×5 mL of PBS with 0.1% DDM. Finally, chimeric C100Flag substrate was eluted by competition with a flag peptide (Sigma, Inc.) diluted in PBS containing 0.1% DDM.

### *In vitro* cleavage assay

Chimeric membrane protein substrates (1.5 µM) were mixed with detergent-purified MCMJR1 (4 µM) in a final volume of 25 µL in PBS at pH 7.4 containing 0.1% DDM, and in the presence of 4 µg/mL of E-64, 14 µg/mL of Bestatin and 100 µM of PMSF. The reaction was allowed to continue for 8 hours at 37°C. Proteolytic activity generated a N-terminal product (∼46 KDa) containing the MBP-tag and a C-terminal product carrying the His_6_-tag (∼4 KDa). Detection of the former was achieved directly by either coomassie blue staining of a 10% SDS-PAGE gel or by immunoblotting with anti-MBP specific antibodies (NEB, Inc).

### Identification of the cleavage sites by mass spectrometry

The chimeric substrate (10 µM) was incubated at 37°C with MCMJR1 (2 µM) in a final volume of 1 mL in PBS (pH 7.4) supplemented with 0.1% DDM, and in the presence of 4 µg/mL of E-64, 14 µg/mL of Bestatin and 100 µM of PMSF. Following overnight incubation, 500 µL of pre-equilibrated Ni-NTA resin was added to the reaction mixture to remove undigested chimeric substrate and C-terminal cleavage product, both His_6_-tagged. For mass spectrometry analysis, the flow through containing the N-terminal cleavage product was concentrated to 200 µL and precipitated at −20°C with 1 mL of 10% Trichloroacetic acid (TCA) in acetone. The precipitated protein was collected by centrifugation at 17,000× g for 5 minutes and the pellet washed with 1 mL of acetone at −20°C. The pellet was solubilized in 8 M urea and the suspension passed through a 30 KDa cut-off centrifugal device (Millipore, Inc.) to exchange the urea for an ammonium bicarbonate buffer at pH 7.4. The protein was then concentrated to a final volume of 40 µL. Thrombin cleavage was carried with a 20 µL aliquot of the concentrated protein sample with 0.1 U of enzyme (Roche) to release a small peptide defined by the thrombin site at the N-terminus and the MCMJR1 cleavage site at the C-terminus. The N-terminal cleavage products, without and with thrombin treatment were analyzed by MALDI-TOF mass spectrometry using a Voyager DE-STR (Applied Biosystems). Each mass spectrum was averaged from 500 measurements and calibrated with myoglobin as an internal standard. The spectra were smoothed and further analyzed using the software M-over-Z (Genomic Solutions, Inc.).

### Inhibition of MCMJR1

MCMJR1 diluted in dialysis buffer to a final concentration of (0.8 µM) was first pre-incubated for 2 hours at 37°C with γ-secretase transition-state analog inhibitors L-685,458 [15] and 31C [29], [49] purchased form Sigma and Calbiochem, respectively. The final inhibitor concentrations were 10, 25, 50, 75, 100 and 200 µM for L-685,458, and 10, 50, 75 and 100 µM for 31C. The substrate Gurken-TMD (0.8 µM) was added following pre-incubation with the inhibitors and the reaction was quenched with SDS-PAGE sample loading buffer 8 hours later.

## Supporting Information

Figure S1 Activity of MCMJR1 loop deletion variants. A coomassie stained 12% SDS-PAGE analysis of purified (left panel) variants Δ 169-203 (the amino acids 169-203 have been deleted), Δ 169–217, Δ 176–196 and Δ 176–203. The wild-type enzyme was included for comparison. The right panel shows a coomassie stained 10% SDS-PAGE analysis of the incubations of the loop deletion variants with Gurken-TMD. Protein bands corresponding to the undigested (black arrowhead) and digested (white arrowhead) substrates are indicated on the right and the molecular weight marker positions are shown on the left side.
(0.35 MB TIF)

Results S1 Identification of putative GXGD-type diaspartyl intramembrane proteases from archaea.
(0.04 MB DOC)
