# Supplementary figures and images for: Identification of an Archaeal Presenilin-Like Intramembrane Protease
Source: PLoS One. 2010 Sep 29;5(9):e13072. doi: 10.1371/journal.pone.0013072 (PMC2947513; doi:10.1371/journal.pone.0013072)

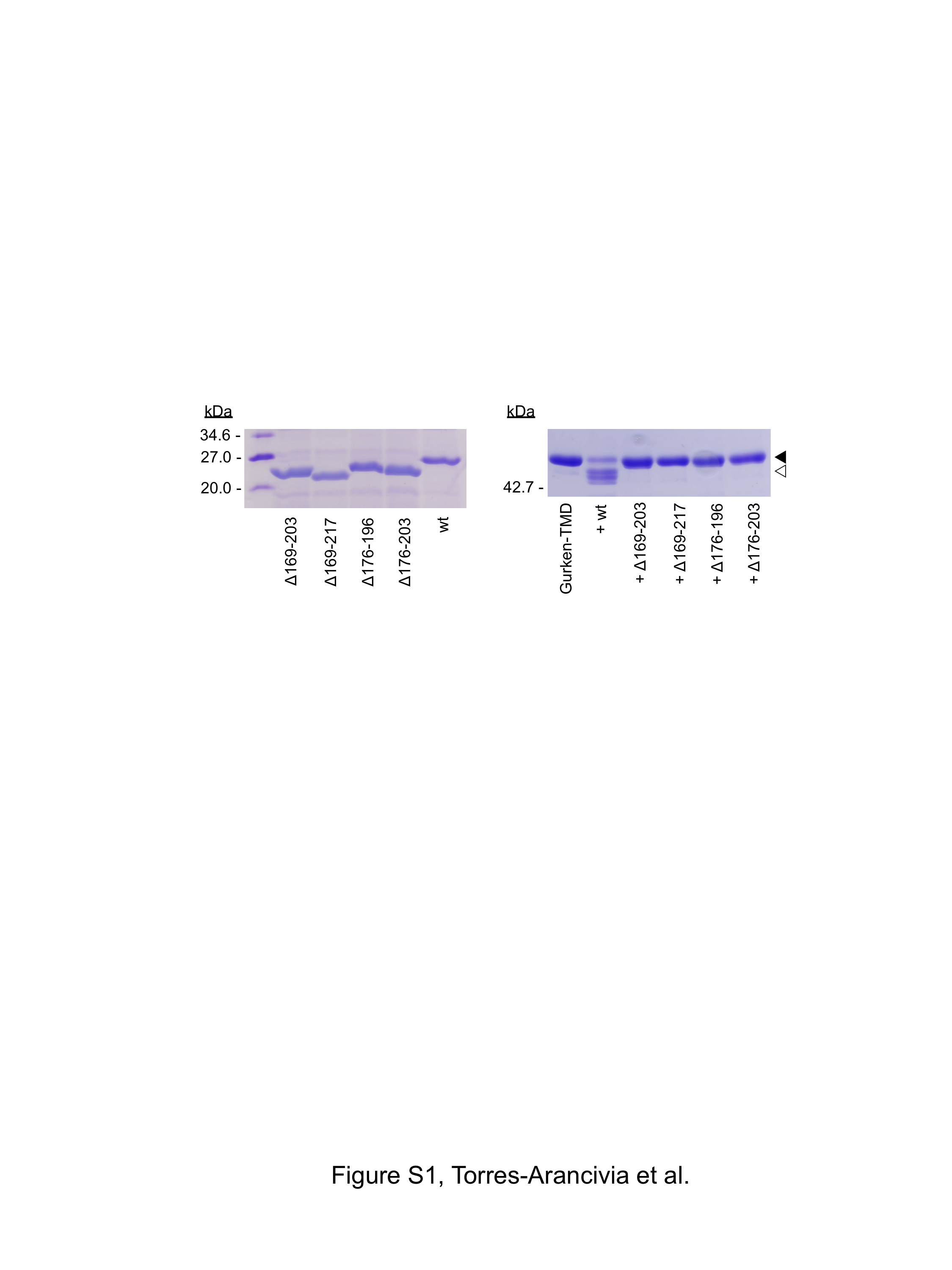

Supplement: Figure S1 — Activity of MCMJR1 loop deletion variants. A coomassie stained 12% SDS-PAGE analysis of purified (left panel) variants Δ 169-203 (the amino acids 169-203 have been deleted), Δ 169–217, Δ 176–196 and Δ 176–203. The wild-type enzyme was included for comparison. The right panel shows a coomassie stained 10% SDS-PAGE analysis of the incubations of the loop deletion variants with Gurken-TMD. Protein bands corresponding to the undigested (black arrowhead) and digested (white arrowhead) substrates are indicated on the right and the molecular weight marker positions are shown on the left side. (0.35 MB TIF) [file pone.0013072.s001.tif]
