# Supplementary material for: Identification of an Archaeal Presenilin-Like Intramembrane Protease
Source: PLoS One. 2010 Sep 29;5(9):e13072. doi: 10.1371/journal.pone.0013072 (PMC2947513; doi:10.1371/journal.pone.0013072)
Supplement: Results S1 — Identification of putative GXGD-type diaspartyl intramembrane proteases from archaea. (0.04 MB DOC) [file pone.0013072.s002.doc]

**Results S1**

**Identification of putative GXGD-type diaspartyl intramembrane proteases from archaea**

Despite limited areas of direct sequence homology [1], presenilins and SPPs share a multispanning membrane topology and identical YD and GXGD signature motifs, carrying the two catalytic aspartates on adjacent TMDs, as well as a PAL motif near their C-termini. We used these signature motifs as seeds to carry out a simple genomic expansion into all known archaeal genomes using BLASTP [2]. From this search 12 putative GXGD-type diaspartyl intramembrane proteases that were confined to commercially available archaeal genomes were selected and cloned. The accession numbers and origin of the identified sequences were YP_134827.1 from *Haloarcula marismortui*, NP_632933.1 from *Methanosarcina mazei*, NP_070776.1 from *Archaeoglobus fulgidus*, [ZP_05570178.1](http://www.ncbi.nlm.nih.gov/protein/257075817?report=genbank&log$=prottop&blast_rank=37&RID=7FYY7DNX01P)from *Ferroplasma acidarmanus*, YP_023524.1 from *Picrophilus torridus*, [YP_503938.1](http://www.ncbi.nlm.nih.gov/protein/88603760?report=genbank&log$=prottop&blast_rank=3&RID=7FYY7DNX01P) from *Methanospirillum hungatei*, NP_110537.1 from *Thermoplasma volcanium*, YP_001047832.1 from *Methanoculleus marisnigri*, [NP_393541.1](http://www.ncbi.nlm.nih.gov/protein/16081237?report=genbank&log$=prottop&blast_rank=33&RID=7FYY7DNX01P) from *Thermoplasma acidophilum*, [YP_304267.1](http://www.ncbi.nlm.nih.gov/protein/73668252?report=genbank&log$=prottop&blast_rank=7&RID=7FYY7DNX01P) from *Methanosarcina barkeri*, YP_566542.1 from *Methanococcoides burtonii* and [NP_618870.1](http://www.ncbi.nlm.nih.gov/protein/20092795?report=genbank&log$=prottop&blast_rank=8&RID=7FYY7DNX01P) from *Methanosarcina acetivorans*.

**References**

1. Ponting CP, Hutton M, Nyborg A, Baker M, Jansen K, et al. (2002) Identification of a novel family of presenilin homologues. Hum Mol Genet 11: 1037-1044.

2. Altschul SF, Gish W, Miller W, Myers EW, Lipman DJ (1990) Basic local alignment search tool. J Mol Biol 215: 403-410.
